# Exploring the role of spending on malaria incidence in Uganda using the auto-regressive distributed lag approach

**DOI:** 10.1186/s12936-024-04929-8

**Published:** 2024-04-30

**Authors:** Jemimah Katushabe, John Bosco Nnyanzi, Gertrude Sebunya Muwanga

**Affiliations:** School of Economics, Makerere University, Kampala, Uganda

**Keywords:** Malaria Incidence, ARDL, Health expenditure, Uganda

## Abstract

**Background:**

Malaria has remained a persistent global health problem. Despite multiple government and donor initiatives to eradicate malaria and its detrimental effects on Uganda's health outcomes, the incidence of malaria is worrying as it appears higher than the average of 219 cases per 1000 for sub-Saharan Africa for the period 2017–2018. This study investigated the effect of public and private healthcare spending on the incidence of malaria in Uganda.

**Methods:**

Employing time series data spanning over 20 years from the first quarter of 2000 to the last quarter of 2019, the study builds a model based on the Grossman framework for analysing demand for health. The estimation technique used was the ARDL approach that takes into account reverse causality and incidental relationships. Prior to the adoption of the technique, a bounds test was performed to determine whether the variables contained in the model have a long-term relationship. Several diagnostic tests for serial correlation, functional normality, and heteroskedastic specification error were carried out to verify the ARDL model's goodness of fit. Additionally, the cumulative sum of recursive (CUSUM) and cumulative sum of squares of recursive residuals (CUSUMSQ) were used to test model stability.

**Results:**

The results indicate that in the long run, an increase in public spending of one percent significantly reduces malaria incidence by 0.196 at the 10 percent level of significance. On the other hand, there is no significant evidence of private health expenditure's effect on malaria incidence. However, in the short run, public spending reduces malaria incidence by a smaller magnitude of 0.158 percent relative to the long-run. Still, private expenditure is found to exhibit no significant effect. Additional findings point to the importance of GDP per capita and urban population growth in reducing malaria incidence, whereas female unemployment, income inequality, as well as female-headed household. In the short run, however, the female-headed households and urban population growth are found to significantly reduce malaria incidence while an improvement in regulatory quality decreases malaria incidence by 0.129 percent.

**Conclusions:**

There is need for further government interventions to reduce malaria incidence in the country via budget allocation, as well as the strengthening of programmes to raise household income to support private health spending, in addition to the development of strategies to promote well-planned and organized urban centres.

## Background

Malaria has remained a persistent global health problem. Children and pregnant women are particularly at risk for malaria, making it a major global public health concern. In 87 countries where malaria is endemic, there were 229 million estimated cases in 2019, down from 238 million in 2000. There were 218 million estimated malaria cases worldwide in 2015, according to the baseline data for the Global Technical Strategy for Malaria (GTSM) 2016–2030 of the World Health Organization (WHO) [1]. Global malaria incidence decreased from 80 cases per 1000 people at risk in 2000 to 58 cases in 2015 and 57 cases in 2019. Global malaria case incidence decreased by 27 percent between 2000 and 2015, and by less than two percent between 2015 and 2019, showing a slowing of the decline's pace since 2015 [2]. The 2022 WHO World Malaria Report further puts the global malaria death to about 619,000 in 2021, which reflects a 9 percent increase from the 568,000 deaths recorded before the pandemic struck. Similarly, worrisome is that malaria cases reached 247 million in 2021 compared to 245 million in 2020 and 232 million in 2019 [3].

Understanding the drivers of malaria incidence can be handy in guiding policies aimed at reducing malaria. This is the main objective of the current study. The focus is on Uganda as a country with one of the highest global burden of malaria cases with over 90% of the population at risk, but also a country where malaria remains the leading cause of death, especially in children. According to the WHO [3], there were an estimated 13 million malaria cases and over 19,600 estimated deaths in the country in 2021 alone. Existing data further shows that the disease causes immense detrimental health effects and is responsible for 30 to 50% of outpatient visits and 15 to 20% of hospital admissions [4]. In addition to its considerable impact on morbidity and mortality, the economic sector has not been spared in terms of unimaginable economic loss that has averaged over $500 million during the last decade.

Therefore, malaria continues to rank among Uganda's most critical diseases. Pregnant women and children under five are disproportionately impacted. According to hospital data [5], malaria is thought to be the cause of 30 to 50 percent of outpatient visits, 15 to 20 percent of hospitalizations, and 9 to 14 percent of inpatient mortality. Uganda has the third largest global burden of malaria cases, that is, five percent of the 229 million cases worldwide, and the eighth highest level of deaths, that is, three percent of the anticipated 405,000 malaria-related deaths worldwide [1].

**Fig. 1:**
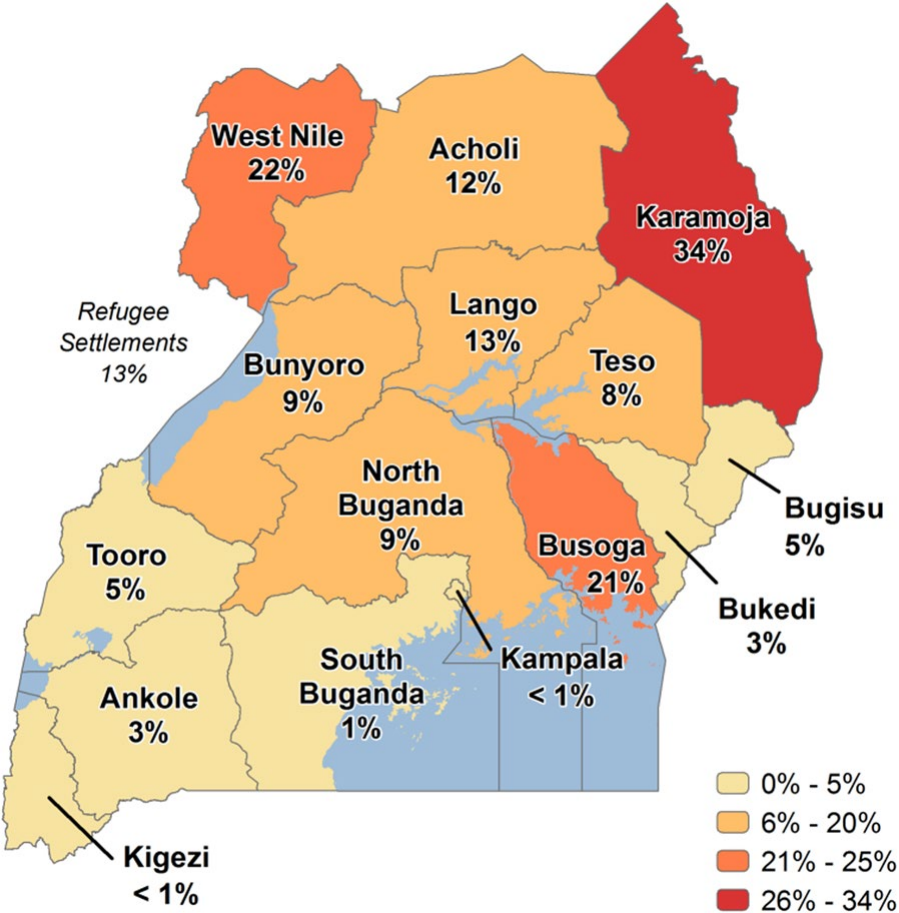
Malaria Prevalence Map—Uganda. Source: Malaria Indicator Survey 2018–2019 [12]

Specifically, the objective is first to determine if government expenditure on health results in low malaria incidence in Uganda. Secondly, the study determines if private expenditure on health results in low malaria incidence in Uganda. Despite multiple government and donor initiatives to eradicate malaria and its detrimental effects on Uganda's health outcomes, the incidence of malaria is worrying as it appears higher than the average of 219 cases per 1000 for sub-Saharan Africa for the period 2017–2018, for example. In fact, Uganda was estimated to have the 3rd highest number of *Plasmodium falciparum* malaria cases globally in 2018, with incidence rates of greater than 250 cases per 1000 population at risk within a perennial transmission setting [6]. According to the 2018 WHO report [7], Uganda is one of the 15 nations that account for 80 percent of the global malaria burden and one of the five that account for nearly half of all malaria cases worldwide. Figure 1 shows malaria prevalence map of Uganda for the period 2018–2019. The map below shows the percentage of children 6 to 59 months of age tested using microscopy who are positive for malaria.

As argued elsewhere [6], given the increasing progress in international efforts to reduce malaria transmission, it is increasingly important to track changes in malaria incidence rather than prevalence. Note that while malaria prevalence reflects the number of existing cases of a disease, malaria incidence reflects the number of new cases of the disease and can be reported as a risk or as an incidence rate. Given the nature of the research question to answer in the current study, as earlier explained, the focus is on incidence, to trace the effect of expenditure on new cases within a specific period. Understanding the drivers of malaria incidence can be handy in guiding policies aimed at reducing malaria cases in Uganda. The purpose of the study is to ascertain how public and private spending affects the incidence of malaria in Uganda to be able to inform policy formulation and implementation.

From the empirical arena, certainly much has been documented albeit with emphasis on the impact of public and private health expenditure on health outcomes, including *inter alia* life expectancy, infant mortality, under-five mortality, or a combination of these factors. One important note from these existing works of literature is that the focus has mainly been on other countries rather than Uganda, but also most importantly, there is scanty literature that considers malaria incidence despite its importance in understanding the dynamics of malaria. Also, a critical methodological issue not given much attention in the previous literature is endogeneity. One related study [8] examines the impact of private and governmental health spending on health indicators (viz. Life expectancy, infant mortality, and the under-five mortality rate inter alia) in 105 countries with moderate- and high-income levels, using the Generalized Least Squares (GLS) technique. The findings showed that public health spending increased health status and had a significant impact on health indicators across all groups. The study found no evidence of the role of private health spending on the selected outcomes.

On the other hand, a study focusing on Nigeria, and utilizing the ordinary least square (OLS) approach, find that while private health spending, the number of doctors, and life expectancy had negative relationships with neonatal mortality rate, child mortality rate, and infant mortality, respectively, government health expenditure per capita had a positive relationship with each of these variables [9]. Similarly, a positive relationship between public health expenditure and life expectancy is reported in a recent study on Ghana during the period 1980 to 2014, draw its findings based on the ordinary least squares (OLS) and two-stage least squares (2SLS) estimators and observes that raising public health spending by 10 percent prevented 0.102 to 4.4 infant and under-five deaths for every 1000 live births while boosting life expectancy by 0.77 to 47 days each year [10]. The latter findings are not much different from a related study employed the Kenyan household data supplemented with county-level data [11].

The study findings based on the ARDL model, which takes care of endogeneity, indicate that in the long run, an increase in public spending of one percent significantly reduces malaria incidence by 0.196 at the 10 percent level of significance. An increase in private health expenditure does not significantly influence malaria incidence at the 10 percent level of significance, but at the 15 percent level of significance, a one percent increase would lead to 0.018 percent decrease in malaria incidence. However, in the short run, public spending reduces malaria incidence by 0.158 percent while private expenditure has no impact.

## Narrative history of malaria health expenditures in Uganda

Uganda's overall public health spending has been rising, but it still falls short of the 15 percent Abuja target and the five percent WHO Commission on Macro Economics target. Similarly, with the current level of health expenditure, Uganda is still far from achieving the 2030 Sustainable Development Goal (SDG) aim to "end the epidemics of AIDS, TB, malaria, and neglected tropical diseases; tackle hepatitis, water-borne infections, and other communicable diseases”. The clear observation is that in general, over the years, the nation continues to be heavily dependent on aid from outside, which accounts for around 45 percent of health spending and raises worries about sustainability. The National Health Accounts (NHAs) for 2018 showed that donor financing for the malaria programme was falling, but there is no evidence that the government is substituting its funds for those of the donors. On the other hand, out-of-pocket health expenses account for 37% of total household consumption spending, much beyond the threshold of 20 percent for catastrophic household consumption spending that is often advised [7].

Specifically, while government funding for malaria programming increased from FY2010/11 to FY2014/15, it decreased in absolute terms in FY 2015/16, from USD 30.6 million (UGX 102,900 million) in FY2014/15 to USD 29 million (UGX 97,400 million). However, it increased as a percentage of the government budget for the corresponding fiscal years, from 21.36 to 21.94 percent. The Global Fund and the U.S. President's Malaria Initiative Uganda (PMI), which increased their disbursement for malaria programmes in Uganda from approximately USD 71.3 million (UGX 239.6 billion) in 2014 to approximately USD 125 million (UGX 420 billion) in 2016, are the two main external malaria funders [7]. Despite the claimed increase in donor financing, households continue to make a sizable financial contribution to the fight against malaria in Uganda, endangering their ability to get effective treatment.

To lower the incidence of malaria, the government is stepping up its control efforts, such as increasing the use of indoor residual insecticide spraying (IRS), long-lasting insecticidal nets (LLINs), and artemisinin-based combination therapy (ACT). Despite this, there are still many cases of malaria. For example, in 2019, there were 263 cases per 1000 persons annually, down from 283 in 2016 [2]. When compared to the target reduction of 50 percent in 2025, this is a pitiful 7.2 percent drop [12]. Available statistics also indicate that in 2020, Uganda had the 3rd highest global burden of malaria cases and deaths (5.4%). This qualified the country to have the 5th highest proportion of malaria cases in East and Southern Africa (23.2%), increasing expenditures to curb the same notwithstanding. This raises the question of whether increasing public and private expenditure on health effectively leads to decreases in malaria incidence.

## Methods

### Data type and source

The study used quarterly time series secondary data for the period 2000:1 to 2019:4. The variables included malaria incidence, public health expenditure, private health expenditure, GDP per capita growth, female unemployment, poverty proportion, urban population growth, female-headed households, foreign aid per-capita, female literacy rate, GINI index, regulatory quality, corruption control, and government effectiveness. Data for all variables was obtained from World Development Indicators [13].

### Model specification

The study employed the Grossman [14] framework for analysing demand for health. The emphasis is on investment in human capital (health and education) for better health outcomes and economic growth. According to the framework, a theoretical health production function can be specified as:

1 $$H = F(X)$$

where H is a measure of individual health output and X is a vector of individual inputs to the health production function F. The elements of the vector include nutrient intake, income, consumption of public goods, education, time devoted to health-related procedures, initial individual endowments like genetic make-up, and community endowments such as the environment.

Although Grossman designed this theoretical model for the analysis of health production at the micro level, several studies including the above-mentioned, have tried to employ his specification at the macroeconomic level. To switch from micro to macro analysis, without losing the theoretical ground, the elements of the vector X were represented by per capita variables and regrouped into sub-sectoral vectors of economic, social, and environmental factors as:

2 $$h = f(y,s,v)$$

where $y$ a vector of per capita economic variables is, $s$ is a vector of per capita social variables, and $v$ is a vector of per capita environmental factors. In its scalar form, Eq. (4) can be rewritten as

3 $$h = f(y_{1} ,y_{2} ,...,y_{n} ,s_{1} ,s_{2} ,...,s_{m} ,v_{1} ,v_{2} ,...v_{l} )$$

where h is the individual’s health status,$\left({y}_{1},{y}_{2},\dots , {y}_{n}\right)=y$; $\left({s}_{1},{s}_{2},\dots , {s}_{m}\right)=s$; $\left({v}_{1},{v}_{2},\dots , {v}_{l}\right)=v$, while $n$, $m$, and $l$ are the number of variables in each sub-group, respectively.

The theoretical model presented above can be modified to health production functions for estimating the relationship between health expenditure and malaria incidence. Due to the existing cultural and environmental conditions of Uganda, and the availability of continuous, reliable, and sufficient data on the variables; the variables representing economic factors are limited to include GDP per capita growth $({y}_{1})$; domestic general government health expenditure per capita $({y}_{2})$; domestic private health expenditure per-capita $({y}_{3})$; female unemployment rate $({y}_{4})$; proportion of population pushed below poverty line $({y}_{5})$; foreign aid per-capita $({y}_{6})$; and GINI index (${y}_{7}$). The variables representing social factors are limited to education proxied by school enrolment at the primary of female as percentage of gross enrolment $({s}_{1})$; female-headed households $({s}_{2})$; regulatory quality $({s}_{3})$; control of corruption $({s}_{4})$; and government effectiveness $({s}_{5})$. The variables representing environmental factors include urbanization proxied by urban population growth $({v}_{1})$; and use of insecticide-treated bed nets $({v}_{2})$; and individual’s health status proxied by malaria incidence $(h)$. The relationship can be expressed as follows.

4 $$mi_{t} = \beta_{0} + puhe_{t - i} + pvhe_{t - i} + \delta_{j} + Z_{t} + \varepsilon_{t}$$

where, ${mi}_{t}$ is the malaria incidence at time $t$, $j=1,...,N$ is the number of economic, social and environmental control variables. According to the WHO [15], malaria incidence refers to the number of new malaria cases per 1000 population at risk per year. In turn, the population at risk is defined as the population living in areas where malaria transmission occurs. ${puhe}_{t}$ represents public expenditure on health at time $t$ and ${pvhe}_{t}$ represents private health expenditure at time $t$ and these two are the variables of prime interest. ${Z}_{t}$ is a vector representing economic, social and environmental control variables, and ${\delta }_{j}$ is a vector of coefficients for economic, social, and environmental variables, while ${\varepsilon }_{t}$ and ${\beta }_{0}$ are the error term and intercept, respectively.

The variables in Eq. (6) were transformed using the natural logarithmic form of the series to capture the elasticities while removing any skewness in the data. The non-linear and non-monotonic link between the independent variables and dependent variables is captured by the model's logarithmic transformation. Heteroscedasticity is also decreased by log transformation. Additionally, the transformation of the variables implies that the coefficients of the variables are read as measuring elasticities. So, the logarithmic transformation results in:

5 $$lnmi_{t} = \beta_{0} + lnpuhe_{t - i} + lnpvhe_{t - i} + \delta_{j} + lnZ_{t} + \varepsilon_{t}$$

It is paramount to do unit root tests of all the variables in the model before estimating the model to avoid the problem of spurious results which emanate from estimation using non- stationary time series. The study employs both Augmented Dickey Fuller (ADF) [16] and Phillips Perron (PP) [17] tests on each of the variables included in the model to ascertain whether they are stationary or non-stationary and to indicate their order of integration [18].

### Estimation technique

The results from the unit root tests, as later shown in Table 5, are indicative of a mixture of integration of I (0) and I (1), suggesting the use of the Pesaran and Shin technique [19]. Specifically, the Bound Testing Cointegration method using the ARDL methodology was used in this investigation to determine if a long run equilibrium relationship exists among the variables. Nevertheless, before estimating the ARDL model, the best lag needs to be determined. The Akaike Information Criterion (AIC) with minimum value or the Schwarz Bayesian Information Criterion (SBIC) might be used for this. The ARDL model used in this study is chosen by SBIC because according to Pesaran and Smith [20] a model selected by SBIC is a more parsimonious model which helps in saving degrees of freedom especially in studies with small sample size like the current study.

Before the ARDL model is estimated, a bounds test must be performed to determine whether the variables contained in the model have a long-term relationship. The following is the ARDL estimate model:

6 $$\begin{gathered} \Delta lnmi_{t} = \alpha_{0} + \alpha_{1} lnmi_{t - 1} + \alpha_{2} lnpuhe_{t - 1} \hfill \\ \quad + \alpha_{3} lnpvhe_{t - 1} + \delta_{j} Z_{t - 1} + \sum\nolimits_{i = 1}^{m} {\alpha_{4i} } \Delta lnmi_{t - i} \hfill \\ \quad + \sum\nolimits_{i = 1}^{m} {\alpha_{5i} } \Delta lnpuhe_{t - i} \hfill \\ + \sum\nolimits_{i = 1}^{m} {\alpha_{6i} } \Delta lnpvhe_{t - i} + \sum\nolimits_{i = 1}^{m} {\delta_{j} Z_{t - i} } + \varepsilon_{t} \\ \end{gathered}$$

where ∆ = First-difference operator; ${\alpha }_{0}$ = the intercept, $i$ = lag length, ${\varepsilon }_{t}$ = error term, $j=1,...,N$ is the number of economic, social and environmental control variables. The Wald test (F-statistics) is employed to determine whether there is a long-term link between the variables in the model. The alternative hypothesis for the test asserts that cointegration among the variables in the model does exist, contrary to the null hypothesis, which argues that cointegration among the variables in the mode does not exist. The following is a statement of the null and alternative hypotheses:

$$\begin{gathered} H_{0} :\alpha_{1} = \alpha_{2} = ... = \alpha_{i} = 0 \hfill \\ H_{a} :\alpha_{1} \ne \alpha_{2} \ne ... \ne \alpha_{i} \ne 0 \hfill \\ \end{gathered}$$

The calculated F-statistic is compared to the critical F-values given by Pesaran, Shin, and Smith [21], where two sets of critical values, that is, lower bound critical value and upper bound critical value, for a particular significance level are provided. For all classes of the variables, such as strictly I (0), strictly I (1), or mutually cointegrated, they yield boundaries critical values.

Based on an F-statistic bound table and the following criteria, cointegration is concluded. The null hypothesis is rejected, implying that there is cointegration between the variables if the estimated F-statistic is greater than the higher critical value. Note that it cannot be concluded that there is no cointegration if the estimated F-statistic is less than the lower bound critical value. The test is inconclusive if the estimated F-statistic is between the lower and upper boundaries, and a determination of the existence of a long-term association requires information about the order of integration.

A diagnostic test for serial correlation, functional normality, and heteroskedastic specification error were carried out to verify the ARDL model's goodness of fit. The cumulative sum of recursive (CUSUM) and cumulative sum of squares of recursive residuals (CUSUMSQ) were used to test model stability.

The ARDL model's ECM equation for this investigation is as follows:

7 $$\begin{gathered} \Delta lnmi_{t} = \gamma_{0} + \sum\nolimits_{i = 1}^{m} {\gamma_{1i} } \Delta lnmi_{t - i} + \sum\nolimits_{i = 1}^{m} {\gamma_{2i} } \Delta lnpuhe_{t - i} + \sum\nolimits_{i = 1}^{m} {\gamma_{3i} } \Delta lnpvhe_{t - i} \\ + \sum\nolimits_{i = 1}^{m} {\delta_{ji} Z_{t - i} } + \gamma_{4} ECT_{t - 1} + \mu_{t} \\ \end{gathered}$$

where ECT is the error correction term, $\mu_{t}$ is the error term.

$lnmi$ is the logged annual incidence of malaria cases per 1000 at-risk populations. In this study, it served as the dependent variable. Public health expenditure per capita ($lnpuhe$) in logs, is the amount the government spends on healthcare expressed in current international US dollars at purchasing power parity. $lnpuhe$ and $lnmi$ are expected to be negatively correlated, a priori.$lnpvhe$ stands for current private health expenditures per capita expressed in US current international dollars at purchasing power parity and is in log form. $lnpvhe$ is included as a separate determinant variable because public allocations do not account for private health spending, even though they make up a sizable share of all SSA health expenditures. $lnpvhe$ affects $lnmi$ via augmenting $lnpuhe$, making necessary medications affordable, and people using dietary supplements. The expected sign is negative. Table 1 provides the variable description and source of data.

**Table 1:** Variable description and source of data

Variable	Description	Source
$$lnmi$$	Incidence of malaria (per 1000 population at risk)	World Bank WDI
$$lnpuhe$$	Log of Domestic general government health expenditure per capita, PPP (current international $)	World Bank WDI
$$lnpvhe$$	Domestic private health expenditure per capita, PPP (current international $)	World Bank WDI
$$ggdppgr$$	GDP per capita growth (annual %)	World Bank WDI
$$lnue\_fem$$	Unemployment, female (% of female labor force) (modeled ILO estimate)	World Bank WDI
$$lnpov\_prop$$	Proportion of population pushed below the $1.90 ($ 2011 PPP) poverty line by out-of-pocket health care expenditure (%)	World Bank WDI
$$urbanpopn\_gr$$	Urban population growth (annual %)	World Bank WDI
$$lnfemhh$$	Female headed households (% of households with a female head)	World Bank WDI
$$lnodapc$$	Net ODA received per capita (current US$)	World Bank WDI
$$rqlty$$	Regulatory Quality (WB WGI Estimate)	World Bank WGI
$$ccorr$$	Control of Corruption (WB WGI Estimate)	World Bank WGI
$$litracy\_fem$$	Literacy rate, adult female (% of females ages 15 and above)	World Bank WDI
$$geff$$	Government Effectiveness (WB WGI Estimate)	World Bank WGI
$$lngini$$	Log of GINI index. A measure of income inequality	World Bank WDI

The variables contained in the vector $Z_{t}$ arise from literature and include; GDP per-capita growth ($ggdppgr$), female unemployment ($lnue\_fem$), poverty proportion ($lnpov\_prop$), urban population growth ($urbanpopn\_gr$), female-headed households ($lnfemhh$), foreign aid per-capita ($lnodapc$), female literacy rate ($litracy\_fem$), GINI index ($lngini$), regulatory quality ($rqlty$), corruption control ($ccorr$) and government effectiveness ($geff$). The rationale for including these variables can be found in the next section.

$ECT_{t - 1}$ is the lagged error correction term of the residual from the cointegrating regression equation. It captures the adjustment toward the long-run equilibrium. The coefficient ${\gamma }_{4}$ represents the proportion of disequilibrium in malaria incidence in one period corrected in the next period. That is, the speed of adjustment, and is expected to have negative sign.

## Results

### Summary statistics, rationale for variable inclusion and the pairwise correlation matrix

The fundamental descriptive statistics for all variables by the original data are shown in Table 2. The lowest malaria incidence was 250.62 in the third quarter of 2015, and the highest was 509.68 in the third quarter of 2001, indicating a wide range in malaria incidence within the sample. It was also discovered a lot of heterogeneity in the health-care spending proxies. With a mean of $16.66, public health expenditures ranged from $12.006 (second quarter of 2000) to $22.918 (fourth quarter of 2010). With a mean of $42.57, private health expenditures ranged from $18.89 (first quarter of 2004) to $59.945 (third quarter of 2013). The lowest percentage population increase in urban centres (5.763 percent) occurred in the first quarter of 2000, while the highest occurred in the third quarter of 2017 (6.257 percent). The summary statistics show reduced levels of skewness and kurtosis, indicating that data normality is not a problem. The minor discrepancies between the mean and median values of these variables support this, implying that the data has a high level of consistency.

**Table 2:** Summary statistics (N = 80): Original variables

Variable name	St. dev.	Mean	Median	Min	Max	Skewness	Kurtosis
Malaria incidence	83.641	385.25	414.83	250.62	509.68	− 0.239	1.744
Public health expenditure	3.265	16.66	16.113	12.006	22.918	0.551	2.06
Private health expenditure	13.833	42.57	47.096	18.89	59.945	− 0.444	1.673
Urban population growth	0.14	5.92	5.865	5.763	6.257	1.244	3.281
GDP per capita growth	2.14	2.81	2.702	− 0.907	7.68	0.352	2.387
Female unemployment	0.855	3.285	3.194	2.011	4.707	0.137	1.38
Poverty proportion	0.503	3.10	3.103	1.65	4.05	− 0.815	4.192
GINI index	1.056	43.11	42.947	40.914	45.249	− 0.047	2.534
Foreign aid	7.529	45.99	46.138	28.385	59.307	− 0.333	2.834
Female head households	1.233	28.85	29.091	25.213	31.444	− 0.601	3.211
Female literacy rate	4.246	62.80	62.598	53.388	71.099	− 0.056	3.436
Corruption control	0.113	− 0.938	− 0.918	− 1.252	− 0.776	− 0.371	2.286
Government effectiveness	0.077	− 0.516	− 0.528	− 0.632	− 0.363	0.412	1.874
Regulatory quality	0.104	− 0.18	− 0.219	− 0.44	0.046	0.467	2.706

The inclusion of GDP per-capita growth as a measure of an increase in average income per person in a specific country serves as a control variable for utilization of healthcare services since income is an influencing factor in health-seeking behaviour. A priori, it would be expected that this variable to be negatively correlated with malaria incidence rates, based on the argument that as real per capita increases, one would expect the standards of living of the people to improve. Similarly, the female unemployment rate (the proportion of the female population ages 15–64 that is not economically active) was included to capture the effect of female unemployment on home health production for family members or how their absence in the labor market denies supplementing family income to command more health inputs to help improve the health of their families [22]. The expected sign is positive. Additionally, poverty proportion quantifies the percentage of the population that out-of-pocket medical expenses cause to fall below the $1.90 ($2011 PPP) poverty threshold. This variable is included because people with different socioeconomic statuses experience illness burden, coverage, and impact of public health interventions differently. To be more specific, the poorest of the poor are disproportionately affected by malaria [23]. Moreover, as the latter authors state, in developing nations, the poorest populations frequently reside in the most isolated places and are ostracized socially or culturally. Therefore, a priori, the variable is expected to be positively correlated with malaria incidence rates.

The other variable appearing in the analysis is urban population growth (i.e. urban population growth as a percentage of total population growth). For, as some authors have argued [24], urbanization rate may improve the health status since health facilities in urban areas are more cost-effective. Moreover, it is avered that in Africa, malaria transmission is comparatively higher among the rural setting than urban areas which may be because of the higher vector density, lower housing quality, and the poor drainage systems in rural settings [25]. Therefore, a prior expected sign for this variable is negative. Also included is the female-headed household measured as percent of households with a female head. This is meant to illustrate how gender roles affect how households control malaria. Recently, it has been argued that female-headed families are more likely to report adopting preventative measures against malaria, such as dousing the home in pesticide, draining stagnant puddles, and maintaining a clear environment [26]. However, this may not always be true. The expected sign of the coefficient is therefore uncertain. Foreign aid is another control factor included in our model, measured as the official development assistance provided per person in current US dollars. The rationale for its inclusion is because, as earlier presented, external donors provided the vast majority (95%) of financing for malaria prevention, control, and treatment. A negative sign is expected.

Similarly, the study includes female literacy (the ratio of female to total primary school enrollment, regardless of age, to the population of the age group that officially corresponds to the level of education shown) because it captures the efficiency with which health is produced, and reduces the malaria incidence rates [27]. This is because in most developing countries, women play an important role in family health such as sleeping under mosquito-treated nets and contributing to malaria eradication programmes [24], and therefore, women’s ability to make informed health decisions for the family could be enhanced by their literacy level since educated mothers are more likely to be aware and assimilate information about the health needs of the family [28]. An a priori expectation is that female literacy is negatively related to malaria incidence. Income inequality, as measured by the GINI coefficient, is also included in the analysis as it is alleged that higher-income households are more likely than lower-income households to undertake malaria prevention measures [29]. As such, it is hypothesized that the variable would possess a positive sign. The data source for all the aforementioned variables is the World Bank World Development Indicators [13].

However, institutional variables, with data sourced from the World Bank World Governance Indicators [13] are also included. Specifically, regulatory quality is included because poor-quality and counterfeit pharmaceuticals pose a serious risk to the public's health by directly raising the likelihood of treatment failure, the development of antimicrobial resistance, morbidity, death, and healthcare costs. When a socially disadvantaged group or groups within a population are unable to reach their optimal level of health, health inequalities result. For instance, when those with lower socioeconomic statuses are more likely to suffer from poor health outcomes, it leads to an unequal distribution of health that may be traced back to a specific social situation. Moreover, it has been argued that the burden of malaria falls disproportionately on children, the poor, and rural communities in low- and middle-income countries [30]. The regulatory quality is expected to be negatively correlated with malaria incidence rates.

The second institutional variable is the Control of Corruption that tracks the misuse of public funds allocated for the fight against malaria for personal gain. In essence, corruption depletes resources, making it difficult to provide for the needs of the community, such as access to clean water, good health care, and education [31]. A priori, corruption control is expected to be negatively correlated with malaria incidence rates. Finally, government effectiveness is another institutional factor included in our analysis as it is likely that in developing nations the government plays a crucial role in the provision of high-quality healthcare, implying that government efficiency is critical. For, a government's capacity to develop and carry out sound policies and provide for the common good is measured by its effectiveness. Moreover, as elsewhere suggested [32], the policies that result from competent government management include *inter alia* health insurance, free prenatal care, and good road infrastructure. As a result, the incidence of malaria is directly correlated with government effectiveness. A negative correlation between the two variables is expected.

The check for regressors’ correlation (to exclude strongly correlated variables) revealed no issues (correlation coefficients were found to be below 0.8, as suggested by econometric studies). The correlation coefficients between the independent variables are shown in Table 3. Multicollinearity was unlikely to be a problem in the estimations, as the coefficients were relatively low. As a result, no explanatory variables were removed from the study.

**Table 3:** Pairwise correlation of explanatory variables

Vars	(1)	(2)	(3)	(4)	(5)	(6)	(7)	(8)	(9)	(10)	(11)	(12)	(13)
(1)	1.00												
(2)	0.59	1.00											
(3)	− 0.05	0.14	1.00										
(4)	0.22	− 0.25	0.25	1.00									
(5)	0.19	0.55	0.40	− 0.38	1.00								
(6)	− 0.53	− 0.62	0.40	0.45	− 0.26	1.00							
(7)	0.20	0.74	0.19	− 0.21	0.69	− 0.36	1.00						
(8)	− 0.18	− 0.12	− 0.35	− 0.54	0.12	− 0.09	− 0.05	1.00					
(9)	0.11	0.44	0.31	− 0.08	0.70	− 0.04	0.62	0.26	1.00				
(10)	0.14	0.45	− 0.03	− 0.47	0.56	− 0.36	0.54	0.61	0.55	1.00			
(11)	− 0.28	− 0.28	0.58	0.44	0.17	0.55	0.04	− 0.47	0.17	− 0.51	1.00		
(12)	− 0.21	− 0.65	0.13	0.39	− 0.38	0.47	− 0.52	− 0.24	− 0.49	− 0.68	0.47	1.00	
(13)	− 0.16	− 0.73	0.01	0.64	− 0.61	0.60	− 0.59	− 0.26	− 0.37	− 0.75	0.52	0.76	1.00

(1) public health expenditure, (2) private health expenditure, (3) GDP per capita growth, (4) female unemployment, (5) poverty proportion, (6) GINI coefficient, (7) foreign aid per capita (8) urban population growth, (9) female-headed household, (10) female literacy rate, (11) corruption control, (12) government effectiveness, (13) regulatory quality. All variables are in logs with the exception of GDP per capita growth

### Unit root tests results

The results of the stationarity tests based on the Augmented Dickey-Fuller (ADF) and Phillips-Peron (PP) unit root tests are shown in Table 4. The stationarity tests show that at 95 percent level of confidence, the data are not stationary with the exception of GDP per-capita growth, the percentage of people living in poverty, the growth of the urban population, and female headed households which are stationary at the level. Thus, the variables are either I (0) or I (1).

**Table 4:** ADF and PP unit root test results

Variable	ADF			PP		
	statistic (Z_t_)	p-value	Order of integration	Test-statistic (Z_t_)	p-value	Order of integration
Malaria incidence—level	− 1.224	0.6630		− 0.124	0.9469	
Malaria incidence—1st Diff	− 3.381	0.0116**	I(1)	− 3.959	0.0016***	I(1)
Public health exp.—level	− 2.082	0.2518		− 1.598	0.4845	
Public health exp.—1st Diff	− 3.696	0.0042***	I(1)	− 4.625	0.0001***	I(1)
Private health exp.—level	− 2.094	0.2467		− 1.677	0.4427	
Private health exp.— 1st Diff	− 3.439	0.0097***	I(1)	− 4.254	0.0005***	I(1)
GDP per capita growth—level	− 3.505	0.0079***	I(0)	− 2.625	0.0880*	I(0)
Female unemployment—level	− 2.527	0.1089		− 1.572	0.4978	
Female unemployment— 1st Diff	− 4.653	00001***	I(1)	− 4.245	0.0006***	I(1)
Poverty proportion—level	− 2.963	0.0385**	I(0)	− 5.125	0.0000***	I(0)
Urban population growth—level	− 5.027	0.0000***	I(0)	− 1.503	0.0532	I(0)
Female headed HH—level	− 3.246	0.0175**	I(0)	− 3.178	0.0213**	I(0)
Foreign aid—level	− 2.241	0.1918		− 1.637	0.4638	
Foreign aid—1st Diff	− 3.943	0.0017***	I(1)	− 4.763	0.0001***	I(1)
Female literacy—level	− 2.187	0.2111		− 1.295	0.6313	
Female literacy—1st Diff	− 3.553	00067***	I(1)	− 3.401	0.0109**	I(1)
GINI—level	− 2.652	0.0827*	I(0)	− 1.181	0.6818	
GINI—1st Diff				− 3.173	0.0216**	I(1)
Regulatory quality—level	− 1.499	0.5338		− 1.214	0.6673	
Regulatory quality—1st Diff	− 3.809	0.0028***	I(1)	− 4.715	0.0001***	I(1)
Corruption control—level	0.325	0.9785		1.060	0.9949	
Corruption control—1st Diff	− 3.125	0.0247**	I(1)	− 3.083	0.0278**	I(1)
Government effectiveness—level	− 3.304	0.0147**	I(0)	− 2.205	0.2045	
Government effectiveness—1st Diff				− 4.798	0.0001***	I(1)

All variables in logs except GDP per capita growth, urban population growth and the governance quality. Diff. is difference; exp. is expenditure

### Bounds test results

Based on the bounds test, when the computed F-statistic is greater than the upper bound, I (1), the null hypothesis that no cointegration exists between the series is rejected. However, if the F-statistic is less than the lower bound, I (0), the null hypothesis that there is no cointegration between the series is accepted. Otherwise, if the F-statistic falls between I (0) and I (1), our inference would be inconclusive. The findings across all models show that for I (0) regressors, the null hypothesis of no cointegration is rejected at one percent. Additionally, for I (1) regressors, all models show that the null hypothesis is rejected at a one percent level. It can therefore be concluded that there is a long run relationship at the one percent significance level except Model 3 for which it exists at the five percent significant level. The results of the bounds test are presented in Table 5.

**Table 5:** Results for Pesaran, Shin, and Smith (2001) bounds F-test

	Model 1		Model 2		Model 3		Model 4	
	Lower I(0)	Upper I(1)	Lower I(0)	Upper I(1)	Lower I(0)	Upper I(1)	Lower I(0)	Upper I(1)
1% level	2.79	4.10	2.65	3.97	2.65	3.97	2.65	3.97
5% level	2.22	3.39	2.14	3.30	2.14	3.30	2.14	3.30
10% level	1.95	3.06	1.88	2.99	1.88	2.99	1.88	2.99
F-Statistic	4.427		4.254		3.996		4.828	

The null hypothesis is ‘no level relationship’; Critical values from Pesaran/Shin/Smith (2001) are presented

## ARDL model estimation results

### Effect of public and private expenditure in the longrun and shortrun

In Table 6, Model 1, (the preferred model), the main finding is that in the long run, increasing public spending by one percent would cause malaria incidence to reduce by 0.196 percent. This result is statistically significant at five percent level. Also, in terms of magnitude, public expenditure on health yields greater effect in terms of reducing the malaria incidence compared to private spending. Inferentially, government health spending, in contrast to findings recorded elsewhere [33], is crucial for lowering the incidence of malaria in Uganda. These results are in line with past empirical findings [8, 32, 34], *inter alia]*. It can however be noted that private health expenditure does not significantly influence malaria incidence in the long run. This could point to the possibility that many Ugandans suffering from malaria turn to using local herbs rather than spend money on malaria treatment. Nevertheless, the private health expenditure effect on malaria incidence is, as expected, negative, although it was not statistically significant [coefficient = − 0.018, p-value = − 0.18]. By implication, the possibility for the private spending increase to reduce malaria incidence exists.

**Table 6:** The long run effect of healthcare expenditure on malaria incidence

Dependent variable: Malaria incidence	(1)	(2)	(3)	(4)
Public health expenditure	− 0.196**	− 0.215**	− 0.184**	− 0.376**
	(− 2.15)	(− 2.31)	(− 2.05)	(− 2.56)
Private health expenditure	− 0.018^**#**^	0.045	0.030	0.127
	(− 0.18)	(0.39)	(0.30)	(1.03)
GDP per capita growth	− 0.464***	− 0.305	− 0.492***	− 0.689***
	(− 2.95)	(− 1.29)	(− 3.12)	(− 3.75)
Female unemployment	0.186***	0.118**	0.185***	0.218***
	(3.82)	(2.24)	(3.84)	(3.30)
Urban population growth	− 0.619***	− 0.577***	− 0.607***	− 0.701***
	(− 3.60)	(− 3.34)	(− 3.56)	(− 3.74)
GINI	0.960**	1.370**	0.995**	1.288**
	(2.02)	(3.83)	(6.62)	(5.16)
Female-head HH	1.947***	1.744***	1.899***	2.250***
	(6.86)	(2.06)	(2.11)	(2.24)
Regulatory quality	− 0.366	0.177	0.413*	0.528*
	(− 1.64)	(0.72)	(1.79)	(1.96)
Female literacy		− 1.048		
		(− 1.39)		
Government effectiveness			− 0.091	
			(− 0.69)	
Corruption control				− 0.015*
				(− 1.88)
Constant	0.544	0.886*	0.564	0.526
	(1.29)	(1.80)	(1.33)	(1.19)
Observations	78	78	78	78
Adjusted R-squared	0.84	0.85	0.84	0.85

Malaria incidence is the dependent variable; All variables are in log form except GDP per capita growth, urban population growth, literacy rate, and the governance quality indices; The ARDL model lags selected by SBIC criterion in the short run as (2,2,0,0,0,2,0,2,1), (2,2,0,0,0,2,2,1,0,1), (2,2,0,0,0,2,2,0,1,0), and (2,2,0,0,0,2,2,1,0,0) for each model respectively; t-values in parentheses; *** p < 0.01, ** p < 0.05, * p < 0.1; while # indicates 15% level of significance (p < 0.15)

On the other hand, the lack of statistical significance could point to the ineffectiveness of the private individuals to take care of themselves when it comes to malaria treatment. To support this argument, recent authors [33] have found out that high out-of-pocket expenses act as a barrier to reducing malaria incidence. This is probably because in Africa, out-of-pocket expense percentage attributed to self-care in terms of disease fight is low. This leads to limiting access to prevention and treatment measures, delayed diagnosis and inadequate treatment. It is not uncommon to find some attributing their sickness to witchcraft. As a result, there is increased malaria transmission and higher incidence rates. Moreover, many Africans suffer from financial constraints that hinder preventive measures such as buying insecticide-treated bed nets and antimalarial medication. When one has nothing to eat, it is very difficult to think of buying a mosquito net or even going in for expensive malaria treatment. The most vulnerable are the poor folks normally living in SSA.

Based on the results presented in Table 7, the short-run malaria incidence effect of public health expenditure remains highly significant at a one percent conventional level, though the coefficient is lower in magnitude. Precisely, increasing public health expenditure by one percent would yield a reduction of about 0.158 percent in malaria incidence in the short-run. Note, however, that at the first lag, the effect turns significantly positive. Nevertheless, the latter finding is not uncommon in literature as can be traced in an earlier study [35] to determine whether health spending reduces malaria cases in Kwara State of Nigeria. Private health expenditure having zero lags did not appear in the short run which shows that it has no impact on malaria incidence in the short run. This may be because the private expenditures are undertaken for treatment purposes other than prevention purposes in the short-run. However, treating malaria in the short run eliminates *P. falciparum* that causes malaria in the population thereby reducing the malaria incidence in the long-run.

**Table 7:** The short run effect of healthcare expenditure on malaria incidence

Dependent variable:$lnmi$	(1)	(2)	(3)	(4)
Malaria incidence (lag 1)	0.581***	0.535***	0.572***	0.418***
	(6.37)	(5.86)	(6.18)	(3.84)
Public health expenditure	− 0.158***	− 0.134***	− 0.162***	− 0.165***
	(− 4.84)	(− 4.11)	(− 4.86)	(− 5.10)
Public health expenditure (lag 1)	0.159***	0.147***	0.155***	0.130***
	(4.21)	(4.03)	(4.02)	(3.39)
Urban population growth	0.820***	0.481**	0.803***	0.597***
	(3.92)	(2.31)	(3.79)	(2.98)
Urban population growth (lag 1)	− 0.809***	− 0.679***	− 0.805***	− 0.646***
	(− 3.48)	(− 3.11)	(− 3.45)	(− 2.95)
Female-head Households	0.924***	0.904***	0.925***	0.916***
	(7.41)	(6.95)	(7.39)	(7.46)
Female-head household (lag 1)	− 0.803***	− 0.771***	− 0.799***	− 0.643***
	(− 5.42)	(− 5.32)	(− 5.37)	(− 4.18)
Regulatory quality	− 0.129**		− 0.129**	
	(− 2.47)		(− 2.46)	
GINI coefficient		− 1.503***		− 0.977***
		(− 3.32)		(− 2.72)
Female literacy		0.787**		
		(2.54)		
$ECT (-1)$	− 0.166***	− 0.168***	− 0.169***	− 0.147***
	(− 4.26)	(− 4.27)	(− 4.30)	(− 3.57)

Lags are chosen automatically based on AIC; t-values in parentheses; ***p < 0.01, **p < 0.05, *p < 0.1. $lnpvhe$, $lnue\_fem$, $lngdppc$, and $lngini$ do not appear because they have zero lags and have no impact in the short run. ECT(-1) is error correction term

### Additional findings

The long-term impact of the GDP per capita on malaria incidence is also demonstrated by the results. GDP per capita coefficient is negative and significant at one percent level in the long run. This suggests that a one percent rise in GDP per capita results in a decrease in the incidence of malaria by 0.464. This finding is consistent with an earlier study [36] where it is asserted that economic growth may lessen malaria if it made more resources available for the disease's prevention. This is conceivable given the high direct expenses associated with treating and preventing malaria. An increase in household income might make it possible for them to spend more on mosquito control measures including providing bed nets, using insect repellents, and emptying wetlands and canals. Increased income would also enable households to spend more on medical care, including medication, travel, professional fees, and lodging at a hospital.

Higher incomes at the national level would make it possible to build public health facilities for the treatment of malaria patients and local initiatives to lessen the number of mosquitoes in a region. Additionally, increasing income is linked to the movement of workers to cities, which may reduce the percentage of the population that lives in rural areas with high rates of malaria. Considering all of these factors while holding constant variables like geography and temperature, it can be anticipated that wealthy countries will have lower malaria rates [36].

The study also found that in the long run, malaria incidence would increase when the fraction of female unemployment rises. The coefficient of female unemployment is statistically significant at one percent conventional level. Malaria incidence would increase by 0.186 percent as the percentage of female unemployment rises by one percent. On the other hand, increasing urbanization appears good news for the control of malaria. According to the results in Tables 7 and 8, the relevant coefficient is significant at one percent and displays a negative relationship between urbanization and malaria incidence both in the long run and short run. Specifically, a one percent growth in urban population would reduce malaria incidence by 0.619 percent and 0.809 in the short run and long run, respectively. Similarly, recent studies [37, 38] noted that infection probability declined with increasing urbanization, suggesting this be due to the reduction of suitable breeding grounds for malaria vectors through the reduction of vegetative cover, water surfaces, and other natural surfaces with building structures and other paved surfaces as well as through pollution of available breeding sites.

**Table 8:** ARDL-ECM model diagnostic tests

Test statistic	Model 1	Model 2	Model 3	Model 4
Durbin-Watson: Lower-order autocorrelation	2.291	2.325	2.291	2.203
Breusch-Pagan LM test: Higher-order Autocorrelation	4.199 (0.240)	5.725 (0.126)	4.378 (0.223)	3.505 (0.32)
Jarque–Bera: Normality test	3.68 (0.159)	5.66 (0.06)	8.95 (0.011)	4.66 (0.097)
White-test: Homoscedasticity, Chi2	78.00 (0.446)	78.00 (0.446)	78.00 (0.446)	78.00 (0.446)
LM test for ARCH	0.747 (0.387)	0.510 (0.475)	0.708 (0.40)	0.045 (0.83)
Ramsey reset	1.51 (0.221)	3.99 (0.121)	2.50 (0.10)	2.17 (0.101)
CUSUM	Stable	Stable	Stable	Stable
CUSUMSQ	Stable	Stable	Stable	Stable

In parenthesis are Chi (2) values for Jarque–Bera, and p-values for other tests

Source: Author own computations

The current study also found that long-term malaria incidence would be impacted by income inequality. The GINI coefficient is positive and significant at a five percent level in the long run. This means that malaria incidence would increase by 0.960 percent as income inequality rises by one percent. This result is consistent with a recent study [39] where it is contended that the long-term effects of inequality become apparent when a high growth rate for vulnerable people with low income is observed. This indicates that rising economic disparity would increase the number of persons who find it difficult to afford both personal protection against malaria and its treatment because this category includes the lowest-income earners.

Relatedly, an increase in the number of female-headed households would lead to an increase in malaria incidence in the long run and short run. Malaria incidence will rise by 0.924 percent in the short term for every one percent increase in the proportion of female-headed households in the short run. When the proportion of households with a female head rises by one percent, malaria incidence will rise by 1.947 percent over the long term. This may be the case since female headed households are among the most vulnerable groups in society and are more likely to live in poverty, which makes it difficult for them to prevent malaria. Regulatory quality would impact malaria incidence negatively, though the impact is not significant.

The ECT coefficient is -0.166, and is significant at one percent level of significance. This demonstrates that any divergence from the long-term equilibrium between the variables and the incidence of malaria may be corrected and regained on average every quarter at a rate of 0.166 percent.

### Diagnostic tests results

In Table 8, a summary of the diagnostic tests that were used to identify model flaws is presented. There was no serial correlation in the models, according to the findings of the Breusch-Godfrey LM Test (BG test) and the Durbin-Watson test. There was no heteroscedasticity issue in the models, as demonstrated by the White test technique and the LM test for ARCH. The anticipated Ramsey reset tests have shown that the estimated models' functional form was correct and they were free of the omitted variables issue. In a similar manner, the results of the CUSUM and CUSUMSQ tests (not shown here due to space but available on request) indicated the model structures were stable. The Jarque–Bera test indicated that the models were normal, except model 3, thus model 1 was the most appropriate model.

## Concluding remarks

Utilizing quarterly data from 2000Q1 to 2019Q4, the study's primary goal was to examine the short- and long-term relationships between malaria incidence, health spending indicators, and the economic and policy control variables in Uganda. The estimated ARDL models indicate that both public and private health spending have a significant impact on malaria incidence. The incidence of malaria is also greatly impacted by growth in GDP per capita. Additionally, while urban population growth, and an increase in proportion of the population falling in poverty may help to reduce the incidence of malaria in the long and short term, respectively, other factors such as an increase in households headed by women, and widening income inequality are likely to increase it. One other important finding is that female literacy appear to increase malaria incidence in the short run, while regulatory quality reduces it.

It is recommended that the Ugandan government implement new policies to boost health spending and strengthen the programmes being implemented to combat malaria, as well as strengthening the existing undertakings such as the President's Malaria Initiative, Home Based Management, the Health Sector Strategic and Investment Plan, the Against Malaria Foundation, Roll Back Malaria, and the Uganda Malaria Reduction Strategic Plan. Also, there is need to design interventions to ensure that a large percentage of the poorest are using effective treatment, insecticide-treated bed nets (ITNs). Similarly, policymakers are urged to devise strategies for enhancing the standard of regulation. For instance, efforts to strengthen and broaden the reach of the Uganda National Bureau of Standards (UNBS) should be well supported to increase their capacity to enforce quality medical and health-related supplies. On the other hand, the development of suitable infrastructural facilities, such as drainage systems that ensure there is no breeding ground for disease vectors, should be backed by policies or master plans given the evident role of urbanization in malaria incidence in Uganda. The study concurs with previous authors [40] that if Uganda is to eliminate malaria there is a need to ensure access to malaria prevention, diagnosis, and treatment as part of universal health coverage, through increased budget allocation.

In the process of investigation, some areas *albeit* outside the scope of the current study but which deserve attention for future studies were observed. For example, a similar analysis would be carried out for the entire East African region using panel data and using simultaneous equation modelling to discover and estimate the effects that are not discernible in pure cross-sections or time series. Additionally, the use of household-level models with survey data, once available, would be another future area of study to estimate the proportion of new money spent on malaria prevention and treatment, and the success of expenditures made to combat malaria. A detailed venture in these areas would provide a critical contribution to policy in kicking malaria out of the region.

## Data Availability

The study uses quarterly data on several variables spanning a period 2000:1 to 2019:4 for Uganda. All data is available from the relevant sources, including the World Bank World Development Indicators and World Bank Governance Indicators, accessible at https://data.worldbank.org

## Abbreviations

ADF: Augmented Dickey-Fuller
AIC: Akaike Information Criterion
AIDS: Acquired Immune-Deficiency Syndrome
ARDL: Auto-regressive distributed lags
GTFAM: Global fund to fight HIV, tuberculosis and malaria
GTSM: Global technical strategy for malaria
HBMF: Home based management of fever
HSSIP: Health Sector Strategic and Investment Plan
ITN: Insecticide-treated nets
MoH: Ministry of Health
NMCP: National Malaria Control Programme
SDG: Sustainable development goal
SSA: Sub-Saharan Africa
WDI: World Bank Development Indicators
WGI: World Bank Governance Indicators
WHO: World Health Organization
